# Successful treatment of acute circulatory failure of unknown cause using critical ultrasound-guided reverse fluid resuscitation: A case report

**DOI:** 10.1097/MD.0000000000023594

**Published:** 2020-12-18

**Authors:** Haotian Zhao, Ling Long, Zekai Wang, Yaru Yan, Heling Zhao

**Affiliations:** a Department of Ultrasound; b Department of Intensive Care Unit; c Department of Nephrology, Hebei General Hospital; d Department of Ultrasound, The NO.1 Hospital of Shijiazhuang, Hebei, China.

**Keywords:** acute circulatory failure, continuous renal replacement therapy, echocardiography, lung ultrasound, shock

## Abstract

**Rationale:**

Fluid resuscitation manages shock effectively. However, shock is not always caused by hypovolemia; various types of shock have variable volumetric reactivity. Combined echocardiography and lung ultrasound (LUS) is a new technique for assessing volume status and pulmonary edema in these patients. We report a case of unexplained acute circulatory failure and acute kidney injury (AKI) aggravated by active fluid resuscitation. We used the critical consultation ultrasonic examination (CCUE) protocol for evaluation, and successfully revived the patient with reverse fluid resuscitation.

**Patient concerns:**

An 82-year-old man with hypertension, atrial fibrillation, and left ventricular diastolic dysfunction (LVDD) was admitted with abdominal distention and lower extremity edema. He developed symptoms of acute circulatory failure, including low blood pressure, anuria, and skin spots. After positive fluid resuscitation, the blood pressure lowered further, and moist rales were audible over both lungs.

**Diagnosis:**

We performed bedside critical ultrasound for evaluation. The differential diagnoses based on the findings included left atrial and right heart dilatation, low cardiac output owing to reduced left ventricular ejection consequent to excessive circulatory capacity, right heart dilation, and left ventricular compression, and pulmonary edema caused by volume overload.

**Interventions:**

Infusion was withheld, and tracheal intubation and mechanical ventilation were instituted to assist breathing; reverse fluid resuscitation was initiated, using continuous renal replacement therapy (CRRT) to maintain a negative fluid balance.

**Outcomes:**

Within 72 hours of fluid withdrawal, the blood pressure reverted to normal, symptoms of pulmonary edema were alleviated, and the circulation and tissue perfusion were restored. The symptoms of acute renal injury are relieved and allowing urine formation without support.

**Lessons:**

Not all patients with acute circulatory failure require positive fluid resuscitation. Fluid balance should be closely monitored and managed. Potential intolerance to the rapid increase in volume may lead to biventricular interaction, ultimately leading to acute circulatory failure. The shock caused by volume overload should be treated with reverse fluid resuscitation. Combined echocardiography and LUS is a powerful tool for the differential diagnosis of circulatory and respiratory dysfunction.

## 1 Introduction

Acute circulatory failure results from an imbalance of systemic venous return and cardiac function. It causes a decrease in cardiac output, leading to a clinical syndrome of systemic organ insufficiency.^[1]^ Owing to a lack of hemodynamic monitoring equipment in general wards, fluid resuscitation is empirically administered in cases of acute circulatory failure.^[2]^ However, the etiology of shock in certain patients is complex, and various types of shock may coexist. For instance, when hypotension is associated with obstructive (pneumothorax, pericardial effusion, and pulmonary embolism, among others) or cardiogenic (acute left heart failure and myocardial infarction, among others) causes, patients may not respond to fluids despite having a low cardiac output. In these cases, instead of optimizing hemodynamics, fluid resuscitation aggravates the shock. Fluid over-replacement may result in a variety of hazards, including acute kidney injury (AKI) and pulmonary edema among others, which may further deteriorate circulatory function.^[3]^

We report a case of acute circulatory failure in the general ward. After routine rehydration therapy, blood pressure was further lowered and shock was aggravated.

The volume status of the patient was evaluated by combining cardiac, lung, and inferior vena caval ultrasound. After these ultrasound examinations, we considered fluid overload to be the main cause of acute circulatory failure. Bedside critical ultrasound is a powerful weapon for evaluating rapid fluid responsiveness; evaluation may be performed before and after treatment.

Ethical approval was not required for reporting this case. Informed consent was obtained from the patient's family.

## 2 Case presentation

### 2.1 Presenting concerns

The patient was an 82-year-old man, who had a history of hypertension for the past 20 years; his highest recorded blood pressure was 180/110 mmHg, and he took his oral antihypertensive drugs including telmisartan, irregularly. He also had a history of coronary heart disease for >10 years, and was taking oral antiplatelet drugs (aspirin) irregularly. He had experienced a sudden decrease of urine volume for the past 2 days, with abdominal distension and pain, followed by edema of both lower limbs, mental disorder, loss of appetite, and occasional cough and sputum; however, there was no fever or chills.

### 2.2 Clinical findings

The patient was admitted to the emergency department of our hospital, and no urine output was noted after urethral catheterization. Following preliminary investigations, he was transferred to the Nephrology department, where physical examination revealed the following: temperature: 36 °C, heart rate: 105 beats per minute, respiratory rate: 21 breaths per minute, and blood pressure: 145/76 mmHg. The patient was unconscious, and patchy ecchymosis were visible in both arms, forearm, and right groin. The conjunctiva and lips were pale, and the respiratory sounds in both lungs were poorly audible; no wet rales were noted. The heart rhythm was irregular with well audible heart sounds, and the abdomen was distended. Muscular atrophy was noted in both lower limbs with visible tremor in the upper limbs, and the muscle power was of level 4.

### 2.3 Diagnostic focus and assessment

The patient underwent the following tests in hospital on an emergency basis: total protein: 61.73 g/L, potassium: 6.00 mmol/L, sodium: 130.00 mmol/L, chloride: 96.00 mmol/L, anion gap: 23.90 mmol/L, urea: 65.05 mmol/L, creatinine: 756.00 μmol/L, and glucose: 6.70 mmol/L. In addition, blood analysis revealed: leukocyte count: 4.45 × 10^9^/L, neutrophils: 71.70%, lymphocytes: 18.90%, red blood cell count: 3.17 × 10^12^/L, hemoglobin: 103.00 g/L, hematocrit: 0.30, and platelet count: 66.00 × 10^9^/L. The myocardial enzyme levels were as follows: troponin T: 115 ng/L and myoglobin: 354.70 ng/mL. The initial diagnosis in the emergency department was that of acute renal failure, and he was accordingly transferred to the Department of Nephrology. He was finally diagnosed with acute renal injury, atrial fibrillation, coronary artery atherosclerotic heart disease (grade III cardiac function), lung infection, old cerebral infarction, level 3 hypertension (high risk), prostatic hyperplasia (after prostatectomy), urethral stricture, and Parkinson syndrome.

### 2.4 Therapeutic focus and assessment

He was administered diuretics, intravenous immunoglobulin, anti-infectives, and nutritional support at the Nephrology department.

On day 22 of admission, his blood pressure suddenly dropped to 86/55 mmHg, with a heart rate of 107 beats per minute, dyspnea, anuria (urine volume <100 mL/d), abdominal distention, cold extremities, and blood stasis. At this time, he was considered to have shock and insufficient organ perfusion, and was administered rehydration to improve cardiac output. However, the symptoms did not improve after administration of 500 mL of fluid. The symptoms of dyspnea worsened with further decline in blood pressure.

**Figure 1:**
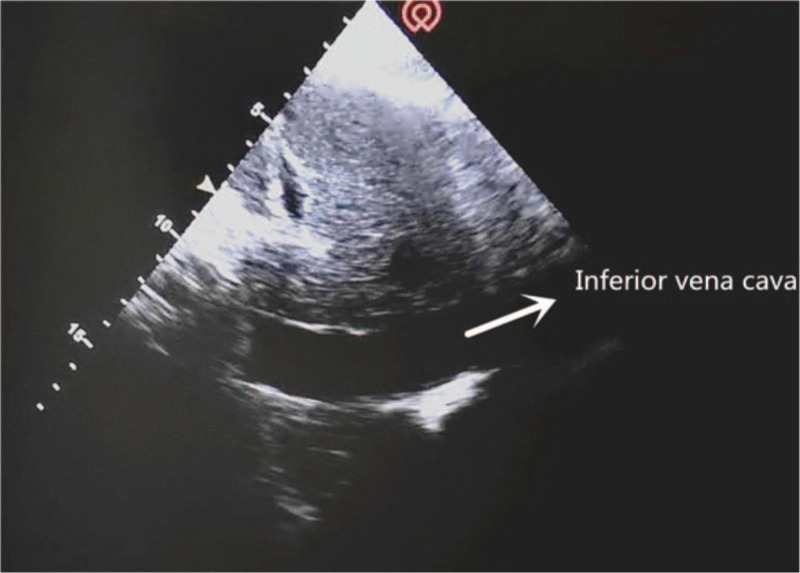
Inferior vena cava.

**Figure 2:**
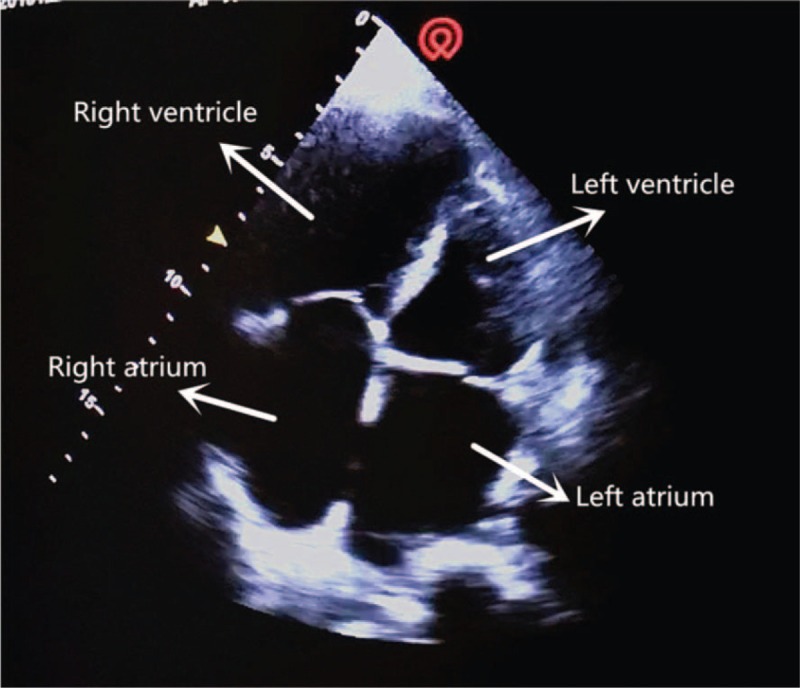
Apical 4 chamber section.

**Figure 3:**
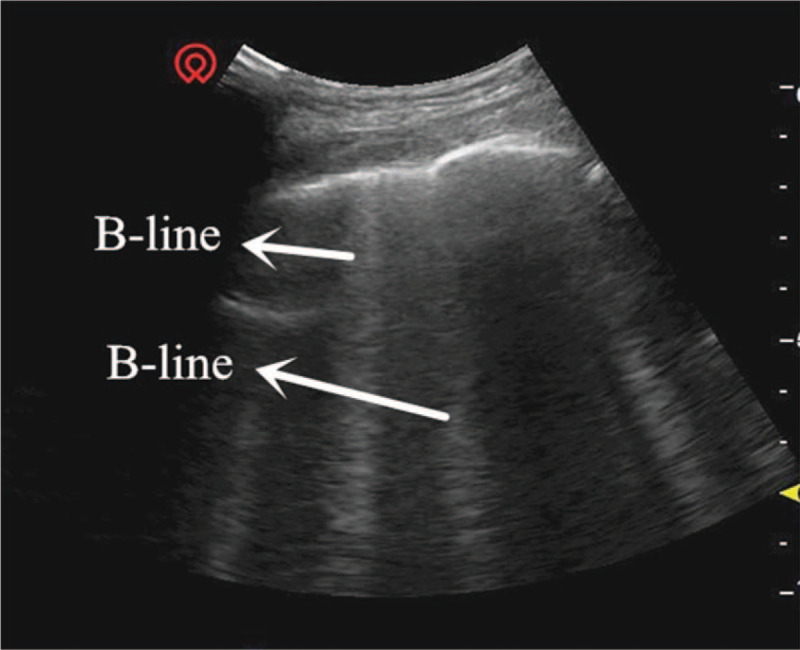
Lung septal rockets.

**Figure 4:**
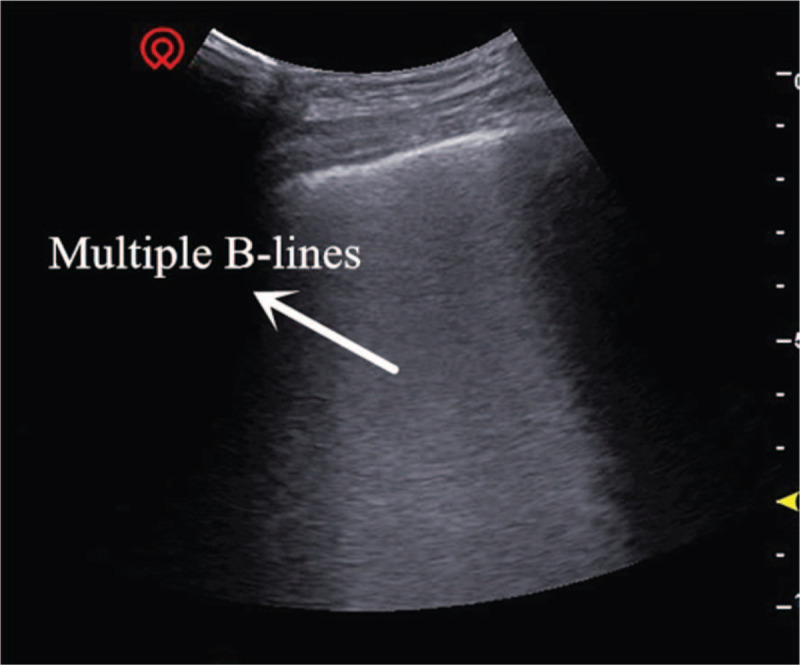
Ground-glass rockets.

**Figure 5:**
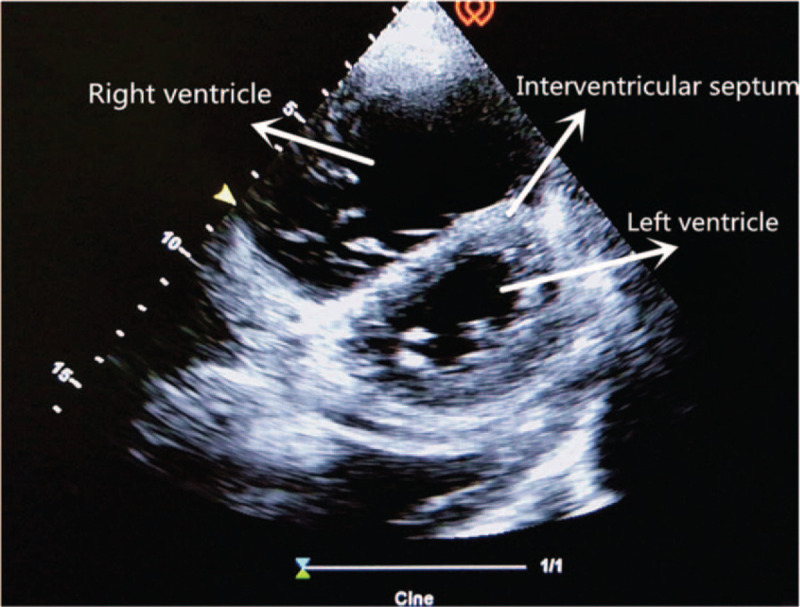
“D shape” of interventricular septum.

The critical-ultrasound specialists were urgently requested to perform the protocol of critical consultation ultrasonic examination (CCUE) at the bedside to evaluate the circulatory and respiratory functions (Tables 1 and 2). The ultrasound examination revealed: a cardiac output of approximately 3.08 L (although the ejection fraction was approximately 65%), widening of the inferior vena cava (Fig. 1) and a low degree of variability with breathing, suggestive of volume overload, increased pulmonary artery pressure with right heart volume expansion (Fig. 2), suggestive of right heart failure, diffuse B-lines in the entire lung, suggestive of severe pulmonary edema (Figs. 3 and 4), and contradictory motion of the interventricular septum, manifested by a rapidly expanding right ventricle squeezing the left ventricle, which results in left ventricular diastolic restriction (Fig. 5).

**Table 1:** Examination results and scores of lung ultrasound in CCUE protocol.

		First time		Second time		Third time	
Lung area	Area of BLUE protocol	Signs of lung tissue	Score	Signs of lung tissue	Score	Signs of lung tissue	Score
Right chest wall	Upper BLUE point	B-lines	1	A-lines	0	A-lines	0
	Lower BLUE point	B-lines	1	B-lines	1	A-lines	0
	Phrenic point	Multi-B-lines	2	B-lines	1	B-lines	1
	PLAPS	Multi-B-lines	2	Multi-B-lines	2	B-lines	1
Left chest wall	Upper BLUE point	B-lines	1	B-lines	1	A-lines	0
	Lower BLUE point	Multi-B-lines	2	B-lines	1	B-lines	1
	Phrenic point	Multi-B-lines Pleural effusion	3	Multi-B-lines Pleural effusion	3	Multi-B-lines Pleural effusion	3
	PLAPS	Multi-B-lines	2	Multi-B-lines	2	B-lines	1
Total scores			14		11		7

**Table 2:** Results of echocardiography and IVC in CCUE.

	Parameter	First time	Second time	Third time
IVC index	End expiratory diameter	28 mm	26 mm	19 mm
	End inspiratory diameter	21 mm	18 mm	12 mm
	Variation with respiration	25%	31%	37%
Cardiac index	Left atrial diameter	60 mm	57 mm	53 mm
	Left ventricular end diastolic diameter	36 mm	41 mm	48 mm
	Right ventricular diameter	45 mm	42 mm	36 mm
	Right atrial diameter	47 mm	46 mm	42 mm
	Left ventricular ejection fraction	71%	65%	64%
	Heart rate	112 bpm	105 bpm	82 bpm
	Cardiac output	≈3.08 L	≈3.47 L	≈4.54 L
	Pulmonary artery pressure	58 mmHg	51 mmHg	46 mmHg
	Abnormal motion of inter-ventricular septum	Presence	Presence	Absence

All the above 5 conditions were indicative of volume overload. Excessive circulating fluid had aggravated the right heart failure and pulmonary edema. Therefore, under the guidance of bedside ultrasound, reverse fluid resuscitation was immediately instituted. Bedside continuous renal replacement therapy (CRRT) was initiated to promote a negative fluid balance. A volume of 800 mL of fluid was removed in 24 hours with CRRT. On the 2nd day, his blood pressure increased to 110/80 mmHg, and the tissue perfusion improved slightly. The CCUE protocol of critical ultrasound was repeated, and CRRT was administered based on the findings. We ensured that the daily fluid output exceeded input. A total volume of 3500 mL of fluid was removed within 72 hours. We can observe that the following time, with the increase of reverse fluid resuscitation, the patient's cardiac load were significantly improved, manifested as the increase of inferior vena cava variability and cardiac output, and the decrease of blood pressure and heart rate. In addition, lung ultrasound (LUS) showed that pulmonary edema also improved significantly, which may be related to the decrease of pulmonary capillary pressure.

### 2.5 Follow-up and outcomes

The circulation stabilized, dyspnea improved, urine output was restored without support and creatinine levels were reduced to 352 μmol/L. The third session of bedside ultrasound showed a marked reduction of B-lines in both lungs, and A-lines appeared in the anterior thoracic region, and the LUS score was decreased (Table 1), suggesting significant improvement of pulmonary edema. Echocardiography showed that the diameter of the left atrium, right ventricle, and right atrium had decreased, while left ventricular end-diastolic volume and inferior vena cava variability had increased (Table 2). This suggested an improvement in his condition, with attainment of stability.

## 3 Discussion

Although the 2016 Sepsis Resuscitation Guidelines recommend fluid resuscitation for patients with confirmed septic shock,^[4]^ Marik and Cavallazzi^[5]^ found that only about half of the patients responded to fluid resuscitation. Therefore, not all cases of hypotension, tissue hypoperfusion, and low cardiac output in septic shock is consequent to insufficient systemic circulatory blood volume. Certain complex shock syndromes may comprise ≥2 types of shock with volume overload. Excessive fluid may not be adequately ejected from the left ventricle and may add to the preload, resulting in increased left atrial filling and pulmonary circulation pressures, pulmonary interstitial or alveolar edema, right heart enlargement, and increased central venous pressures. Pulmonary edema may also be aggravated simultaneously by volume expansion.^[6]^ Central venous pressure (CVP) is the pressure at the end point of venous return. It not only reflects the preload of the heart, but also the afterload of venous return in the systemic circulation. Excessive CVP hinders venous reflux and may increase renal afterload.^[7]^ In cases where the CVP exceeds the renal venous pressure, renal perfusion decreases with aggravation of AKI.^[8]^ Teixeira et al^[3]^ confirmed that fluid balance was an independent predictor of mortality in patients with AKI. In this case, although CVP monitoring was not available in the Nephrology ward, ultrasound monitoring of the inferior vena cava demonstrated the pressure in the central vein and right atrium to a certain extent.

Normally, ventricular ejection is equal on both sides. The right ventricle is less tolerant to afterload pressure (i.e., pulmonary circulatory resistance), so the increase in pulmonary artery pressure due to any reason may cause rapid expansion of the right ventricle. The enlarged right ventricle exerts pressure on the left ventricle through the interventricular septum, leading to the deviation of the interventricular septum towards the left ventricle; this results in the limitation of left ventricular diastolic filling, lowering the stroke volume.

This patient with sudden shock was in a general ward, which lacked hemodynamic monitoring facilities including CVP, pulmonary arterial incarceration pressure, cardiac output, and other tools found in the intensive care unit. Therefore, the knowledge of his hemodynamics was limited. Critical ultrasound is a new technology that has been developed in recent years. It combines the pathophysiological theory of critical diseases (heart failure, respiratory failure, and AKI, among others) with ultrasound technology. It accurately evaluates the circulatory and respiratory status of critically ill patients.^[9]^ The CCUE protocol can differentiate shock types in order to guide treatment. In this case, the patient had signs of circulatory failure including hypotension, skin patches, and oliguria, among others. In the first session of CCUE protocol, both lungs showed diffuse B-lines, suggestive of severe pulmonary edema. Simultaneously, echocardiography showed dilatation of the right ventricle. The systolic force of the right ventricle was significantly poorer than that of the left ventricle, and was inadequate in overcoming the increase of afterload (pulmonary circulation resistance). Therefore, right ventricular enlargement resulted from severe pulmonary hypertension.^[10]^ In addition, in the parasternal short axis section, the enlarged right ventricle compressed the left ventricle through the interventricular septum, restricting left ventricular diastolic volumes. Despite the compensatory increase in left ventricular ejection fraction, the inadequacy in maintaining an adequate cardiac output persists; this may lead to deficient organ perfusion. Assessment of volume responsiveness prior to fluid resuscitation is an important measure in patients with circulatory failure. It is closely related to the choice of follow-up treatment and outcomes. The combined of ultrasound findings of inferior vena cava, heart, and lung with critical ultrasound examination can accurately evaluate the volume responsiveness. Barbier et al^[11]^ found that dynamic monitoring of inferior vena cava variability by ultrasound was an accurate predictor of volume responsiveness. In this case, the inferior vena cava was dilated and fixed, suggesting a high right atrial pressure and a low volume responsiveness.

These ultrasound findings provided evidence of excessive volumes in the cardiopulmonary system; reverse fluid resuscitation was therefore appropriate treatment. In the first 24 hours, the volume state was maintained in negative balance by approximately 800 mL using CRRT. Repeat ultrasound examination showed improvement in several vital parameters (Table 2). It indicated that the treatment was appropriate; however, further close observation was needed. This treatment approach appeared to contradict the traditional concept of treating shock that increases cardiac output via rehydration. It demonstrates the necessity of treating shock based on etiopathogenesis. The essence of treatment in these cases lies in that the same effect has different causes.

Ultrasound is an excellent examination tool in patients who cannot be transported to the imaging department, including older adults, those with multiple organ failure, and those on bedside mechanical ventilation or CRRT. LUS is useful in diagnosing the cause of dyspnea. Lichtenstein performed lung ultrasonography in 6 common diseases causing dyspnea, namely, pulmonary edema, chronic obstructive pulmonary disease, asthma, pulmonary embolism, pneumothorax and pneumonia in the intensive care unit, to confirm its diagnostic accuracy.^[12]^ The diagnosis of pulmonary edema on ultrasound depends on the presence of B-lines. The number of B-lines in 1 positive area (defined as at least 3 B-lines visible between 2 ribs) correlates with the severity of pulmonary edema. If the distance between 2 B-lines (measured at the pleural line) is roughly 7 mm, it may be named “B7-lines,” which correlate with thickened subpleural interlobular septa; these are known as “septal rockets.” If the distance between 2 B-lines (measured at the pleural line) is roughly 3 mm, and several B-lines converge, it correlates with subpleural ground-glass lesions. These are known as “B3-lines” or “ground-glass rockets” and indicate severe interstitial lung disease.^[13]^ The diagnostic value of LUS in pulmonary edema is higher than that of chest radiographs.^[14]^ Gargani et al^[15]^ found that the B-lines appeared earlier on ultrasound than chest radiographs in patients with acute heart failure; this may be used as an early warning indicator. The CCUE protocol integrates cardiac, pulmonary, and inferior vena caval ultrasound to evaluate critically ill patients. It rapidly identifies the cause of acute circulatory or respiratory failure, and evaluates the volume status and responsiveness, guiding further treatment.

## 4 Conclusion

In patients with symptoms of shock including decreased blood pressure, increased heart rate, and oliguria, the volume status should be determined and volume responsiveness should be assessed prior to fluid resuscitation. In critically ill and non-ambulatory patients, bedside ultrasound may provide a clearer and rapid understanding of the patient's condition; it may also allow prompt comparison of treatment outcomes before and after treatment. It may also reduce the risk of other complications, and shows promise in widespread application in the future. Cumulative evidence from further reports is needed to confirm our findings.

## Author contributions

**Resources:** Haotian Zhao, Zekai Wang

**Supervision:** Heling Zhao.

**Visualization:** Haotian Zhao, Ling Long, Zekai Wang.

**Writing – original draft:** Haotian Zhao, Yaru Yan.

**Writing – review & editing:** Ling Long, Heling Zhao.
